# Artificial intelligence and medical education: A global mixed-methods study of medical students’ perspectives

**DOI:** 10.1177/20552076221089099

**Published:** 2022-05-02

**Authors:** Hamza Ejaz, Hari McGrath, Brian LH Wong, Andrew Guise, Tom Vercauteren, Jonathan Shapey

**Affiliations:** 1 Norwich Medical School, 6106 University of East Anglia, UK; 2 Psychological and Behavioural Sciences, 4905 London School of Economics, UK; 3 GKT School of Medical Education, 405987 King’s College London, UK; 4 Department of Neurosurgery, 12228 Yale University School of Medicine, New Haven, Connecticut, USA; 5 Department of International Health, Care and Public Health Research Institute, 4919 Maastricht University, Maastricht, Netherlands; 6 Secretariat, the Lancet and Financial Times Commission on Governing Health Futures 2030, Global Health Centre, 1211 The Graduate Institute, 1211 Geneva, Switzerland; 7 Steering Committee, Digital Health Section, European Public Health Association (EUPHA), 4919 Utrecht, Netherlands; 8 School of Population Health and Environmental Sciences, 111990 King’s College London, UK; 9 School of Biomedical Engineering and Imaging Sciences, 573655 King’s College London, UK; 10 Department of Neurosurgery, King’s College Hospital, London, UK

**Keywords:** Digital, medicine, machine learning, education, automation

## Abstract

**Objective:**

Medical students, as clinicians and healthcare leaders of the future, are key stakeholders in the clinical roll-out of artificial intelligence-driven technologies. The authors aim to provide the first report on the state of artificial intelligence in medical education globally by exploring the perspectives of medical students.

**Methods:**

The authors carried out a mixed-methods study of focus groups and surveys with 128 medical students from 48 countries. The study explored knowledge around artificial intelligence as well as what students wished to learn about artificial intelligence and how they wished to learn this. A combined qualitative and quantitative analysis was used.

**Results:**

Support for incorporating teaching on artificial intelligence into core curricula was ubiquitous across the globe, but few students had received teaching on artificial intelligence. Students showed knowledge on the applications of artificial intelligence in clinical medicine as well as on artificial intelligence ethics. They were interested in learning about clinical applications, algorithm development, coding and algorithm appraisal. Hackathon-style projects and multidisciplinary education involving computer science students were suggested for incorporation into the curriculum.

**Conclusions:**

Medical students from all countries should be provided teaching on artificial intelligence as part of their curriculum to develop skills and knowledge around artificial intelligence to ensure a patient-centred digital future in medicine. This teaching should focus on the applications of artificial intelligence in clinical medicine. Students should also be given the opportunity to be involved in algorithm development. Students in low- and middle-income countries require the foundational technology as well as robust teaching on artificial intelligence to ensure that they can drive innovation in their healthcare settings.

## Introduction

The rise of artificial intelligence (AI) in healthcare has sparked increasing interest in recent years, with promising applications across many areas of medicine.^1^ AI encompasses technologies that learn from data to optimise their work to achieve set goals. In the field of radiology, deep learning (a subtype of AI) has matched the performance of clinicians in the screening for and predicting the risk of lung cancer based on computed tomography (CT) scans,^2^ as well as providing accurate measurements of heart structure and function based on echocardiograms.^3^ In pathology a deep learning algorithm has outperformed a panel of clinicians in the detection of cancerous metastases.^4,5^ During the COVID-19 pandemic, AI has been applied to the interpretation of chest CT imaging with great success, displaying rapid and accurate diagnosis and outperforming radiologists.^6,7^ Multiple reviews point to the potential for AI to transform medical fields such as oncology, radiology and pathology, but also highlight the challenges.^8–10^

The difficulties of implementing AI in clinical settings and the clinical translation gap of AI-based tools are well described, driven in-part by a lack of education and knowledge among clinicians.^11,12^ Interpretability of results and incorporation into cultural constructs within the clinical environment are cited as key obstacles.^13,14^ Furthermore, the trust of clinicians and the general public in healthcare AI has been reduced by high-profile reports of systemic racial biases in widely-used algorithms.^15,16^ These issues may be exacerbated in low- and middle-income countries (LMICs) due to the concentration and development of AI in high-income countries (HICs), despite the growing applications of AI in LMICs.^17–19^ This raises the possibility that clinicians in LMICs will be ill-equipped to utilise AI-based technology to meet the needs of their health systems.

Clinicians, as end-users, are uniquely placed to ensure a patient-centred and equitable roll-out of AI. Expertise in AI among clinicians is not widespread and medical students and allied health students are not taught about AI routinely in medical curricula.^20–22^ There are calls for updating educational practices, with an increased focus on novel digital technologies including AI in order to better prepare medics for work in this changing field.^23-26^ Previous studies have analysed the views of medical students regarding AI in focused samples based in a single institution or country.^21,22,27^ We aimed to collate data from a sample of medical students from around the globe to explore the following questions: (1) What is the knowledge and experience of medical students regarding AI in medicine? (2) What are the AI-related learning needs of medical students? (3) How would they like the medical curriculum to address these needs? Our goal was to provide data that might inform medical educators regarding the needs of future doctors in this emerging space as well as highlight areas where medics may be underprepared for the integration of AI in medical practice. We also hoped to challenge HIC-centred narratives around AI, with contributions from students in LMICs.

## Methods

### Design

We employed a mixed-methods approach for this study by using an online questionnaire and in-person focus groups. The questionnaire provided quantitative data, which was used to ascertain students’ level of knowledge and understanding of AI and the key aspects of AI-related content that should be taught at medical schools. The focus groups provided an opportunity to take advantage of the diverse background and experiences of medical students from different countries and to generate ideas toward the most effective and engaging way to teach AI-related content. Free text data from the questionnaires along with the verbatim focus group data was used to add depth and new perspectives to the quantitative aspects of the questionnaire.^25^

Participants were students of the national member organisations (NMOs) of the International Federation of Medical Students’ Associations (IFMSA). The study received ethical approval under the Low-Risk designation from the King's College London Research Ethics Committee, Review Reference: MRSU-19/20-17484.

### Data collection

The online questionnaire was sent to 140 NMOs through the IFMSA email server a month prior to the 69th IFMSA General Assembly (GA) March 2020 in Kigali, Rwanda. To be eligible for recruitment, individuals had to be a medical student and a member of one of the IFMSA NMOs. They did not need to be a delegate to the March GA. A total of 128 individuals filled out the online questionnaire. Of these, 90 responses were gathered from delegates at the GA recruited through convenience sampling and the remaining 38 were gathered before or after the GA via email.

The questions were adapted from a questionnaire used in a previous study.^21^ It was then reviewed by a team of medical students (HM, HE), qualitative research experts (BW, AG), an AI expert (TV) and a clinician-scientist with expertise in AI research (JS) to ensure internal validity. A copy of the online questionnaire is attached as Supplemental Digital Appendix 1. The questionnaire consisted of the following four sections:

(1) Demographics
(2) What do you learn and know about AI in medicine?
(3) What do you wish to learn about AI in medicine?
(4) How should we change the medical curricula to address these learning needs?

The demographic section collected information regarding participants’ gender, country of origin and country of study. The remaining sections consisted of multiple-choice questions with a combination of four-point Likert scales (Strongly Disagree, Disagree, Agree, Strongly Agree), numerical scales indicating the level of agreement, and binary (Yes/No/Maybe) responses.^26^ At the end of each questionnaire section was a free-text question where participants offered their own thoughts with no word restriction. The questionnaire was handled in accordance with General Data Protection Regulation (GDPR) guidelines in the EU.

Of the 128 participants who filled the questionnaire, 90 attended the in-person focus groups conducted during the GA. Participants were selected for the focus groups through convenience sampling. Those who hadn't already filled out the online questionnaire did so before joining the focus group. The average size of each focus group was 10–15 participants and each was diverse in terms of ethnicity, country of origin, gender, and country of study. Each focus group was led by a trained IFMSA facilitator who obtained written consent from all participants before starting the session. At the beginning of the focus group discussions, we defined the term ‘artificial intelligence’ and we provided examples of uses of AI in fields outside of medicine in order to establish a rudimentary baseline of understanding among participants. The sessions lasted around 90 min each and were divided into discussions around the following two questions:

1. What are the most important aspects of AI to learn about?: Individuals from different countries were paired and given the instruction: ‘Speaking to the person in your pair, come up with five AI-related topics that you consider most important and relevant for medical students to learn’. Once this discussion was completed, pairs joined to form groups of four. These groups discussed their individual lists to come up with a combined set of five topics and ranked them in order of importance. After discussion, the whole group came together to present ideas and discuss salient findings. Data collectors noted the top five topics put forward by each group of four.
2. How should AI be incorporated into the medical curriculum?: A whole group discussion was conducted with the following instructions: ‘In this half of the discussion, we’d like you to share your thoughts regarding how you think AI should be incorporated into your medical curriculum’. The facilitator generally did not intervene in the group discussion unless the discussion was losing focus. Another researcher silently transcribed the discussion.

### Data analysis

Following data collection, data was downloaded from Google Forms to an Excel spreadsheet. Quantitative data from the questionnaire was imported and analysed in R.^27^ The countries of study of the participants were grouped by income status using the World Bank list into LMICs or HICs.^28^ Data visualisation was performed using GraphPad Prism (GraphPad Software, San Diego, California).

All free-text data was copied verbatim into the cells of a spreadsheet. This included free-text data from each section of the questionnaire and data from the open focus group discussion. This free text data was coded by three researchers independently using an adapted version of the framework method.^29^ Each coder went through the data set to come up with their individual set of codes. All coders then discussed the codes and agreed on a set of consensus codes and definitions that were applied to the dataset. Once coding was completed, similar codes were organised under sub-themes and themes. Two iterations through the data were performed: the first was to come up with codes individually and the second was to apply a set of consensus codes.

## Results

A total of 128 medical students from 48 countries participated in the study, with 47 from LMICs and 81 from HICs. There was broad global representation from Africa, Asia, Australasia, Europe, South America and North America. The countries of study with the greatest representation in our sample were India (16.4%), Canada (5.5%) and the United States (4.7%). 52 participants identified as male (41%), 72 participants identified as female (56%) and four students (3%) identified as non-binary. On application of the Framework Method to the free text data,^29^ 28 codes were identified and categorised into 10 general sub-themes (these are provided in the codebook attached as Supplemental Digital Appendix 2). De-identified focus group data, including cohort characteristics, is provided as Supplemental Digital Appendix 3. The quantitative and qualitative results below are organised around the three core research questions.

### What do you know about AI in medicine?

**Figure 1.**
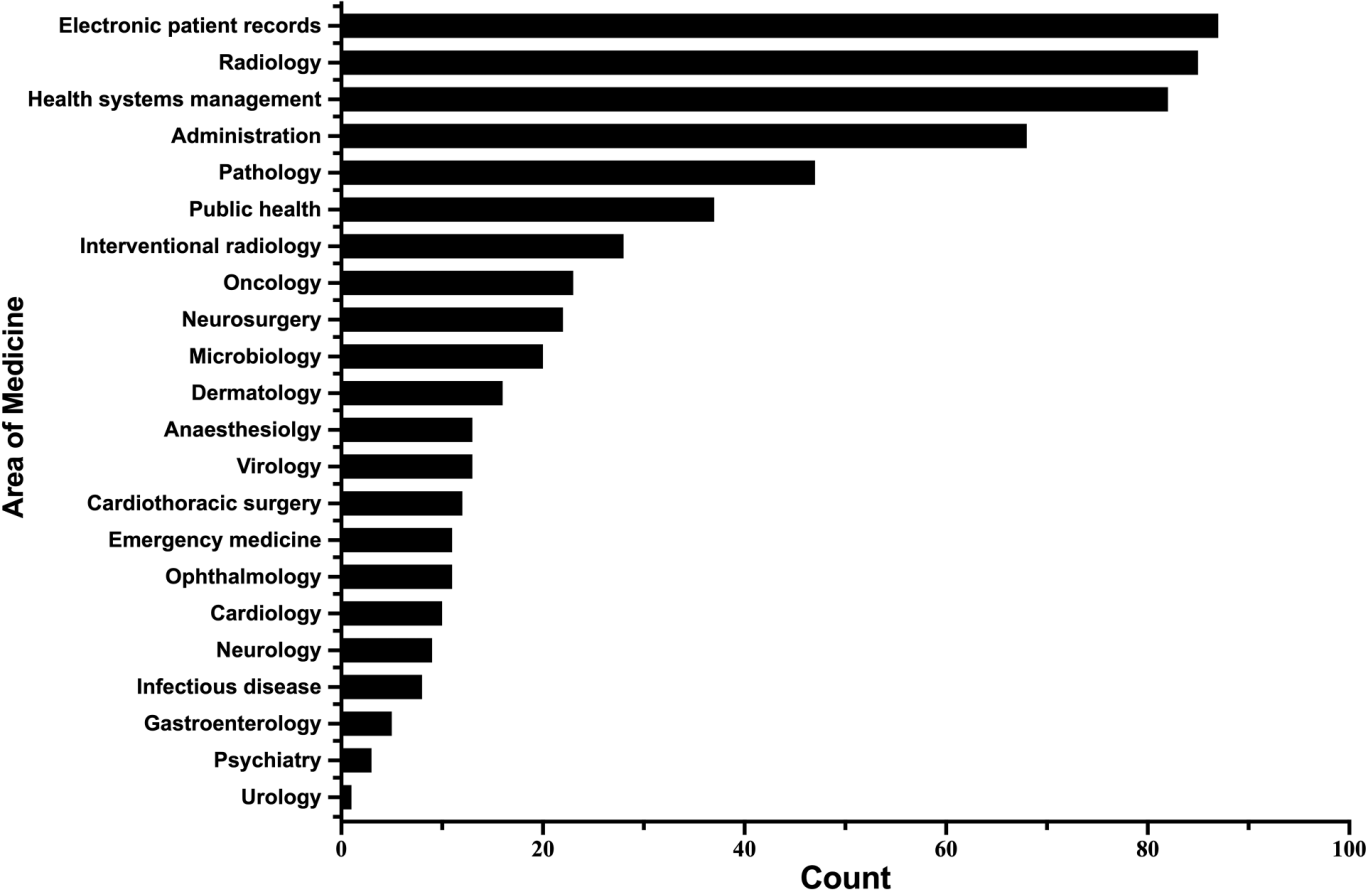
A bar plot of frequencies of students' answers to the question: ‘Which five of the following areas of medicine do you think could be most affected by the introduction of artificial intelligence?’. ‘Health systems management’ explicitly included hospital organisation and scheduling while the label ‘Administration’ included note-taking and secretaries.

The initial questions from the survey focused on assessing the familiarity and understanding of AI among participants. 93 (73%) survey respondents had routine access to electronic patient records (EPR), while 86 (67%) agreed that they were confident in using EPR – indicating a baseline access to and understanding of core healthcare information technologies among the majority. A small minority of students (15.6%) reported coding experience. 93 (73%) respondents had a moderate or strong understanding of the concept of AI, however, only a single student (0.7%) reported significant teaching on AI in medical school. Students believed that the areas of medicine most affected by the introduction of AI would be EPR, radiology and medical administration (Figure 1).

**Figure 2.**
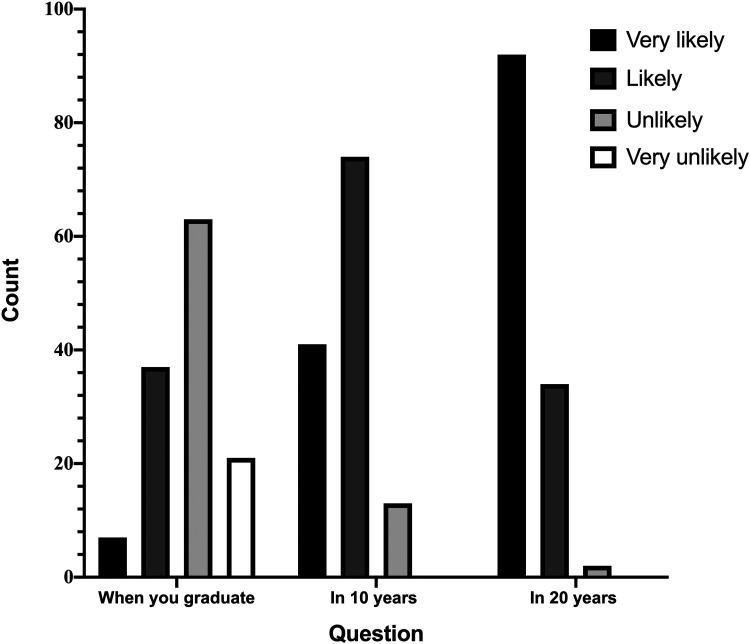
A bar plot of frequencies of students' answers to the question: ‘How likely is it that you will be using AI in your clinical practice in the following time frames?’

The remaining questions of the first section of the survey drew on participants’ opinions and expectations of AI. 120 (94%) respondents agreed with the idea that AI will improve medicine in general, and most students (89.8%) believed that they would be using AI in their work within 10 years (Figure 2). A large proportion (44%) of students felt that AI definitely has a role to play in healthcare in LMICs; 13 (10%) believed that AI definitely does not have a place. Subgroup analysis of the results showed that respondents from HICs were two-and-a-half times more likely to believe that AI is applicable in LMICs compared to respondents from LMICs, indicating a gap in understanding and expectations from students not directly embedded in LMIC healthcare systems. However, 55 (43%) participants reported feeling worried about the impact of AI, and 40 (31%) respondents thought AI will replace physicians and non-interventional doctors in the future.

Qualitative analysis of the free-text responses from the survey and the focus-groups outlined three major sub-themes: applications of AI in clinical medicine, scepticism around AI and applications in LMICs. Applications in clinical medicine include knowledge of the use of AI in general medicine, surgery and diagnosis among others. Student opinion was generally in favour of the integration of AI in clinical medicine, as mentioned by a student from Canada, as it could ‘allow for improvements in health services and their efficiency, freeing up both human and physical resources’. Moreover, students were keen to learn more about AI in their routine medical teaching, particularly its limitations and benefits.

Several students raised ethical concerns and exercised cautious optimism, including a student from Germany who said AI should be ‘mostly used as help … rather than as an executor’ and a student from the UK said that it should be ‘applied to complement and streamline the work of healthcare professionals, but not to replace them’. Students mentioned the potential of AI to improve access to care in underserved communities in LMICs and to improve the quality of care in remote and rural communities. According to a student from Pakistan, ‘it will provide the doctor with the efficient assistance and will save time and money’ in underserved settings. However, a student from Morocco was more sceptical, and suggested that ‘low- and middle-income countries should have access to [foundational] technology first … Some regions still don't have access to the internet’.

### What do you wish to learn about AI in medicine?

Based on the survey responses, the majority of participants (95%) were interested in learning the basic principles of AI, while 61% said they wanted to learn to code. Going further, 103 (80%) agreed that there should be opportunities to get involved in algorithm development and many (91%) felt that doctors should be involved in the integration of AI in clinical practice.

Thematic analysis revealed that students wanted to learn about general concepts and computer science-related theory as part of AI teaching. Salient quotes grouped by sub-themes are presented in Table 1. General concepts incorporate basic principles of how AI works, ethics around the integration of AI in medicine and the skills needed to critically appraise the use of AI. A student from Australia highlighted the need to ‘approach AI as we approach other interventions like drugs or radiology’ and to learn ‘enough about basic principles that we can begin to evaluate for ourselves whether that particular intervention or AI program, is the right choice for our patient’. Another student from Australia raised the concern of AI causing ‘discrimination or unintentional worsening of health outcomes for vulnerable populations’ due to the impact of data bias in AI. They stressed that ‘we should be taught a healthy scepticism for AI … All AI is not created equal, and garbage in = garbage out … we need to be given the knowledge and the tools to make this assessment’. There was a tone of cautious optimism exercised by many students, who expressed that they wanted to learn about the ‘limits and opportunities AI provides to doctors and how they can use AI to do good, and not harm, as a tool’.

**Table 1.** Sub-themes and exemplar quotes generated from analysing qualitative responses to focus group questions. We asked the following: ‘How will AI be applied to medicine in the future? & What would you like to learn about AI that might prepare you for a future in which AI plays an important part in medicine?’ We gathered data from 128 students through online surveys and focus groups carried out at the 69th IFMSA General Assembly in Rwanda, March 2020.

Sub-themes	Exemplar quotes
General Concepts	*‘We should be taught a healthy skepticism for AI … All AI is not created equal, and garbage in* *=* *garbage out … we need to be given the knowledge and the tools to make this assessment.’ – Student from Australia* ‘*The basis of how AI operates, risks and sources of errors in AI … what can AI be used for, what are the limits? What hardware is needed?’ – Student from Germany* *‘What limits and opportunities AI gives us …* *How can we use AI to do good, and not harm, as a tool.’ – Student from Norway*
Applications in clinical medicine	*‘monitoring ICU patient stats … predicting patient outcomes … use of machine learning in order to easily diagnose a patient through imaging … surgical planning and intra-operative guidance.’ – Student from Hong Kong* ‘*within Primary health care… triaging who needs what level of care, follow up on NCDs and other chronic illnesses …* *online consultations’ – Student from Sweden* ‘*detection of diseases at stages where they were previously undetected …* *to predict …* *arthritis in the next five years by looking at … MRIs’ – Student from Pakistan* *it has the potential to recruit massive populations for studies … shown in Apple Heart Study’ –* Student from Ireland ‘*how it can be used with precision’ – Student from Ethiopia*
AI in LMIC	*‘low- and middle-income countries should have access to technology first …* *Some regions still don't have access to internet’ – Student from Morocco* ‘*reach out to the majority of the community’ – Student from Ethiopia* ‘*I also think that AI will be used to increase healthcare access/reach in underserved populations …’ – Student from Australia*
Patient Safety	*‘reduce medical error … identifying …* *skin lesions etc.’ – Student from Ireland*
Computer science	*‘how to code … build AI health related algorithms’ – Student from Burkina Faso* ‘*how the algorithms are developed and trained to understand …* *the results, and also how to mitigate …* *bias.’ – Student from the UK* ‘*the methods used to develop AI algorithms … [to] critically think about the outputs.’ – Student from the United States* *‘how the system integrates and works in the medical system’ – Student from India*
Scepticism	*‘It could be used for general diagnosis … but a health care worker (physician) will still always be required to assess the border line cases ….’ – Student from Iceland* ‘*to ensure that they do not unintentionally cause discrimination or worsening of health outcomes for vulnerable populations … There is great potential for good but also great potential for harm.’ – Student from Australia*
Big Data	*‘How to use technology to stock information, to organize data and to analyse it.’ – Student from Morocco*

Many students wished to learn the basic computer science theory needed to understand AI and some students requested coding classes. A student from Burkina Faso stated they would like to learn ‘how to code …[and] build AI health related algorithms’ while a student from Latvia felt that ‘coding classes should be implemented in the curriculum’. A student from Bangladesh also supported ‘an introduction to coding and a holistic approach to studying AI’ as part of their core medical curricula. A link was drawn by many students between the need to have a basic computer science understanding and applying this to appraise algorithms for clinical translation. Further, a number of participants stressed the need to learn about AI through use in clinical practice, including a student from Japan who felt that students would be more likely to be interested in AI if ‘hospitals introduce AI tech and show its benefit’.

Data from the focus groups showed a ranked order of topics of interest. The most popular topics, in order, were: (1) Applications of AI in clinical medicine, (2) AI 101 including principles and basic coding, (3) AI ethics, (4) AI and the impact on careers in medicine and (5) Algorithm development for clinicians.

### How do you wish to learn about AI in medicine?

The majority (92%) of students who participated in the study stressed that AI-related teaching needs to be incorporated into the core medical curriculum. When asked how they would like AI-related teaching to be incorporated into the curriculum, many students (86%) were interested in exploring interdisciplinary learning with fields including computer science and biomedical engineering.

Qualitative analysis revealed two pertinent themes: core curriculum and teaching delivery. Salient quotes grouped by their sub-themes are presented in Table 2. Most participants agreed on the need to incorporate AI-related teaching into the core curriculum. However, opinions regarding which part of the core curriculum the teaching should be added varied between students. A student from Singapore suggested ‘including more applications of AI during clinical training’ as an integrated course, whereas a student from Russia suggested the need for a ‘specific class on technology and AI in medicine’ during pre-clinical studies. Students from the United Kingdom drew on their experiences with module-based teaching and suggested that ‘each module should have a section about how AI is being and could be used in this field. How to evaluate the effectiveness of AI in a clinical setting and monitor its results’.

**Table 2.** Sub-themes and exemplar quotes generated from analysing qualitative responses to the question: ‘How can the medical curriculum in your country be changed to address the learning needs you have described in this questionnaire?’ We gathered data from 128 students through online surveys and focus groups carried out at the 69th IFMSA General Assembly in Rwanda, March 2020.

Sub-themes	Exemplar quotes
Core Curriculum	*‘basic computer science class … [in] preclinical studies … a specific class on technology and AI in medicine.’ – Student from Russia* ‘*how AI is being and could be used in this field’ – Student from United Kingdom* ‘*Providing an optional curriculum about AI that supports any sort of information around the subject.’ – Student from United Kingdom*
Teaching Delivery	*‘implementing workshops in the university.’ – Student from Belgium* ‘*incorporate actual computer scientists in a multidisciplinary team’ – Student from United Kingdom* ‘*collaboration between medical students, biomedical engineers, computer scientists and data scientists (similar to hackathon-like projects)’. – Student from Hong Kong*
Learning through use	*‘it will never change unless most of hospital[s] introduce AI tech and show its benefit …’ – Student from Japan* ‘*Include more technology in teaching such as lectures and practical sessions so that students can familiarize themselves with the available technology. Hospitals and faculties should adopt AI and technologies as well.’ – Student from Malaysia*

When speaking about teaching delivery, participants stressed on the need for multi-disciplinary learning and hackathon-style workshops. A student from Hong Kong wanted ‘collaboration between medical students, biomedical engineers, computer scientists and data scientists (similar to hackathon-like projects)’ while a student from the United Kingdom supported this idea with the suggestion to ‘incorporate computer scientists in a multidisciplinary team’. A number of students, including one from Belgium, wanted the content to be taught by ‘implementing [AI] workshops in University’.

## Discussion

In this mixed-methods study of medical students across the globe, we found that interest in learning the clinical applications, development and appraisal of AI-based tools was ubiquitous but current medical curricula were not catering to this. Students believed it was important to learn the knowledge and skills necessary to navigate the field of AI in medicine and stressed upon the need for multidisciplinary teaching on the topic.

### Why do students need to know about AI

In the near future, AI is likely to disrupt key areas of medical practice, including psychiatry and diagnostic specialties such as radiology and pathology.^33^ The Topol review in the UK recommended that clinicians should be trained specifically to work with AI in order to ensure improved clinical care.^34^ Previous studies have shown positive results in local and national surveys regarding the knowledge and perspectives of medical students and trainee doctors for an AI-driven future.^21,22^ These studies indicated that, in high-income settings, students were keen to learn more about AI but received minimal specific training on this as part of their medical curriculum. Our study supports the findings of these earlier works: medical students view AI with cautious optimism; they are generally excited for the change that it will bring and they wish to learn more about it. Moreover, our study offers further unique insight into the global distribution of AI-related teaching, experience and interest. Students in HICs and LMICs displayed a broad knowledge of and interest in AI, including its applications in clinical practice as well as the knowledge required to develop algorithms. This is remarkable considering the limited examples of AI-based technologies that have been successfully implemented in clinical practice along with extensive validation and with evidence of improved health outcomes for patients. These findings provide a guide to medical educators willing to address the growing digital renaissance in medicine. Educational institutions can be catalysts for a digital future that is patient-centred if the AI-learning needs of medical students are met.

### AI for mitigating health inequalities

AI offers a unique opportunity to mitigate health inequalities around the globe.^34-37^ AI-based systems have shown promising potential in mitigating the impact of challenges that are specific to LMICs, including high neonatal and maternal morbidity and the spread of Dengue fever.^38–41^ More needs to be done to expand on this success in LMICs specifically. We found extensive interest in AI among students in LMICs but a severe lack of teaching on the topic, and a lack of access to the foundational technology that AI requires. Our data also showed a large gap in expectation between students from HICs and those from LMICs regarding the applicability of AI-based tools to health systems in LMICs given the current infrastructure. Until now, the implementation of AI has been largely focused on high-income settings and socioeconomic groups with high digital literacy.^36,37^ If this trend continues there is the potential for AI to compound the already prevalent issue of health inequality. Further, where AI-based tools are developed for low-resource settings they may not be applicable or appropriate given a HIC-centred approach to development and implementation.^15,38^ Our data supports the notion that teaching around AI needs to be provided to those who are embedded within the systems that will use it, thereby ensuring the local applicability of these tools. According to our data, a good place to start is for healthcare providers in LMICs to be provided the foundational technology to utilise AI in the future. Next, they should be offered the education and resources to bolster AI literacy and to support pioneering advances in the implementation of AI-based tools in their healthcare systems.

### Delivering a new medical curriculum

The need for a multidisciplinary approach towards teaching AI-related content was emphasised by most respondents. Medical schools and other teaching institutions should work together to facilitate these changes to their curricula. A number of institutions are already offering teaching on AI to medical students. Carle Illinois College of Medicine offers a combined medicine and mathematics/data science course with the intention of fostering clinical innovators.^39^ In our study, participants suggested that a major area for change is in content delivery, where AI-related teaching could be given through interprofessional learning between medical and computer science students. Some centres have adopted this approach and one is offering a combined computer science and medicine course in a collaboration between a large hospital and two academic institutions.^24^ Many students suggested hackathon-style or workshop projects, where students collaborate to solve clinical problems, similar to those offered at Harvard Medical School.^40^ This approach would promote creativity as well as improve healthcare and computer science literacy. In terms of organisation, participants suggested that the AI curriculum could be delivered as a separate module, or integrated into current modules with teaching on AI as applied to the relevant specialty. For students who might wish to delve deeper into the topic, undergraduate medical courses could offer an optional, one-year intercalated degree in AI-based healthcare. In postgraduate medical systems, schools should incentivise applicants with a data and computer science background to enter medicine. All of these approaches would address the pressing need for interdisciplinary collaboration and teaching within medical schools.

### Strengths and limitations

The study was designed around data collected at the IFMSA General Assembly due to the unique access to a diverse range of medical students in terms of nationality, socioeconomic status, ethnicity, career stage and clinical and research experience. We used a mixed-methods design for the study as the survey data provided valuable quantitative information on students’ current knowledge and understanding of AI while the focus groups offered an opportunity for students to generate practical ideas about changes to the medical curriculum. Our relatively unbiased sampling is a notable strength of the study – the conference where data was collected was unrelated to AI and technology, and the convenience sampling approach that we took ensured that our participant selection was independent of students’ interest in AI. Offering the survey prior to the focus group provided participants the opportunity to collect their thoughts before the focus group discussions and to maximise the value of these discussions.

We note important limitations with this study that may affect the generalisability of our findings. A key principle in our study was gaining a broad sample of medical students in order to guide educators around the world. In doing so, our sample included a small number of participants from each nation, thereby reducing our ability to reliably associate our findings with particular regions. The geographic distribution of the knowledge, perspectives and wishes of medical students regarding AI in medicine could be an interesting point for future study in order to cater educational interventions to particular regions. We also note the limitations of self-reported data due to its subjective nature. For instance, when ascertaining the understanding of AI among participants, the level of knowledge that one individual defined as a 'poor understanding’ may have been an excellent understanding of AI for another. It would be interesting to explore a more objective understanding among students in the future using questions that test knowledge around AI. The entire study was conducted in the English language in line with the language of the conference. This may have biased study data as participants with a poorer command of English may have been less likely to share their perspectives in group discussion and/or may have articulated these to a lesser extent when answering the questionnaire. While this limitation is difficult to overcome with such a diverse cohort, it could have been mitigated through an offering of (live) multi-lingual translations. Another important point to consider was our impact, as focus group facilitators, on the group discussion. We were mindful of this and hence refrained from expressing our own thoughts on the topic. Instead, our contributions were limited to asking questions that guided and moved the discussion when needed.

## Conclusion

In conclusion, we are in the throes of a fourth industrial revolution, and how doctors adapt to these changes in medicine will determine whether the revolution is patient-centred or not.^41^ Despite the limited clinical implementation of AI-based technologies, medical students wish to be actively engaging with new developments in the field of AI. In order to nurture this interest, they should be provided a holistic core curriculum that offers a basic understanding of the technical aspects of AI as well as its clinical applications. Furthermore, we believe that courses should offer innovative ways to engage students in multi-disciplinary learning with software engineers, computer scientists and other disciplines involved in the development, appraisal, of new algorithms. Evolving medical curricula should be implemented with Health for All values of democracy, equity, solidarity, inclusion, and human rights at its core.^48^ Doing so will not only ensure that new technologies are effective and accessible to those in low- and middle-income settings, fostering a global generation of clinical innovators who can propagate key technologies in the areas of most need, but also safeguard health futures for generations to come.^49^

## Supplemental Material

sj-docx-1-dhj-10.1177_20552076221089099 - Supplemental material for Artificial intelligence and medical education: A global mixed-methods study of medical students’ perspectives Click here for additional data file. Supplemental material, sj-docx-1-dhj-10.1177_20552076221089099 for Artificial intelligence and medical education: A global mixed-methods study of medical students’ perspectives by Hamza Ejaz, Hari McGrath, Brian LH Wong, Andrew Guise, Tom Vercauteren and Jonathan Shapey in Digital Health

sj-xlsx-2-dhj-10.1177_20552076221089099 - Supplemental material for Artificial intelligence and medical education: A global mixed-methods study of medical students’ perspectives Click here for additional data file. Supplemental material, sj-xlsx-2-dhj-10.1177_20552076221089099 for Artificial intelligence and medical education: A global mixed-methods study of medical students’ perspectives by Hamza Ejaz, Hari McGrath, Brian LH Wong, Andrew Guise, Tom Vercauteren and Jonathan Shapey in Digital Health

sj-jpg-3-dhj-10.1177_20552076221089099 - Supplemental material for Artificial intelligence and medical education: A global mixed-methods study of medical students’ perspectives Click here for additional data file. Supplemental material, sj-jpg-3-dhj-10.1177_20552076221089099 for Artificial intelligence and medical education: A global mixed-methods study of medical students’ perspectives by Hamza Ejaz, Hari McGrath, Brian LH Wong, Andrew Guise, Tom Vercauteren and Jonathan Shapey in Digital Health
