# Supplementary material for: Artificial intelligence and medical education: A global mixed-methods study of medical students’ perspectives
Source: Digit Health. 2022 May 2;8:20552076221089099. doi: 10.1177/20552076221089099 (PMC9067043; doi:10.1177/20552076221089099)
Supplement: sj-docx-1-dhj-10.1177_20552076221089099 - Supplemental material for Artificial intelligence and medical education: A global mixed-methods study of medical students’ perspectives [file sj-docx-1-dhj-10.1177_20552076221089099.docx]

Supplemental_Digital_Appendix_1

Artificial Intelligence in Medical Education: Your Views

Thank you for your interest in our study on arti3cial intelligence (AI) in medical education! We coordinated focus groups at the IFMSA 69th General Assembly in Kigali, Rwanda. For those of you who could not join us, we are putting out this questionnaire. We would love to hear your thoughts and ideas on AI!

View our detailed information sheet on the research study here: [https://drive.google.com/3le/d/1hhfogzJs_A1keLXDXq7vH5XC1CbAfRgh/view?usp=sharing](https://www.google.com/url?q=https://drive.google.com/file/d/1hhfogzJs_A1keLXDXq7vH5XC1CbAfRgh/view?usp=sharing&sa=D&source=editors&ust=1615146871436000&usg=AFQjCNGAP84JSziWMoawb6OgYw-XaeIkGw)

The form should only take 10-15 minutes to complete. The three main areas that we cover in this form are:

1. What do you learn/know about arti3cial intelligence (AI)?
2. What do you wish to learn about AI?
3. How do we change the medical curriculum to address these learning needs?

Please don't hesitate to contact us should you have any questions or queries. We can be contacted at: futuramed@gmail.com

We look forward to hearing from you.

Data protection:

By providing your email address, you consent to the use of your personal data in this study. We have tried to minimise the data we collect to that which is necessary. The personal data we collect includes your name, email and country of origin. This data may be used to contact you but we need your consent to do so. All results will be anonymised prior to publication and your personal data will not be included in any of the research output. Your personal data will only be accessible to study personnel to facilitate data collection at the GA. Following the GA all data will be anonymised and personal identi3able data will be destroyed. We will not share any of this information with any third party.

*Required

1. Email address *

Demographic information

1. Full Name *
2. Gender *

Female

Male

Doesn't identify

Other

1. Country of origin *

Afghanistan

Akrotiri

Albania

Algeria

American Samoa

Andorra

Angola

Anguilla

Antarctica

Antigua and Barbuda

Argentina

Armenia

Aruba

Ashmore and Cartier Islands

Australia

Austria

Azerbaijan

Bahamas, The

Bahrain

Bangladesh

Barbados

Bassas da India

Belarus

Belgium

Belize

Benin

Bermuda

Bhutan

Bolivia

Bosnia and Herzegovina

Botswana

Bouvet Island

Brazil

British Indian Ocean Territory

British Virgin Islands

Brunei

Bulgaria

Burkina Faso

Burma

Burundi

Cambodia

Cameroon

Canada

Cape Verde

Cayman Islands

Central African Republic

Chad

Chile

China

Christmas Island

Clipperton Island

Cocos (Keeling) Islands

Colombia

Comoros

Congo, Democratic Republic of the

Congo, Republic of the

Cook Islands

Coral Sea Islands

Costa Rica

Cote d'Ivoire

Croatia

Cuba

Cyprus

Czech Republic

Denmark

Dhekelia

Djibouti

Dominica

Dominican Republic

Ecuador

Egypt

El Salvador

Equatorial Guinea

Eritrea

Estonia

Ethiopia

Europa Island

Falkland Islands (Islas Malvinas) Faroe Islands

Fiji

Finland

France

French Guiana

French Polynesia

French Southern and Antarctic Lands

Gabon

Gambia, The

Gaza Strip

Georgia

Germany

Ghana

Gibraltar

Glorioso Islands

Greece

Greenland

Grenada

Guadeloupe

Guam

Guatemala

Guernsey

Guinea

Guinea-Bissau

Guyana

Haiti

Heard Island and McDonald Islands

Holy See (Vatican City)

Honduras

Hong Kong

Hungary

Iceland

India

Indonesia

Iran Iraq

Ireland

Isle of Man

Israel

Italy

Jamaica

Jan Mayen

Japan

Jersey

Jordan

Juan de Nova Island

Kazakhstan

Kenya

Kiribati

Korea, North

Korea, South

Kuwait

Kyrgyzstan

Laos

Latvia

Lebanon

Lesotho

Liberia

Libya

Liechtenstein

Lithuania

Luxembourg

Macau

Macedonia Madagascar

Malawi

Malaysia Maldives

Mali

Malta

Marshall Islands

Martinique

Mauritania

Mauritius

Mayotte

Mexico

Micronesia, Federated States of

Moldova

Monaco

Mongolia

Montenegro

Montserrat

Morocco

Mozambique

Namibia

Nauru

Navassa Island

Nepal

Netherlands

Netherlands Antilles

New Caledonia

New Zealand

Nicaragua

Niger

Nigeria

Niue

Norfolk Island

Northern Mariana Islands

Norway

Oman

Pakistan

Palau

Panama

Papua New Guinea

Paracel Islands

Paraguay

Peru

Philippines

Pitcairn Islands

Poland

Portugal

Puerto Rico

Qatar

Reunion

Romania

Russia

Rwanda

Saint Helena

Saint Kitts and Nevis

Saint Lucia

Saint Pierre and Miquelon

Saint Vincent and the Grenadines

Samoa

San Marino

Sao Tome and Principe

Saudi Arabia

Senegal

Serbia

Seychelles

Sierra Leone

Singapore

Slovakia

Slovenia

Solomon Islands

Somalia

South Africa

South Georgia and the South Sandwich Islands

Spain

Spratly Islands

Sri Lanka

Sudan

Suriname

Svalbard

Swaziland

Sweden

Switzerland

Syria

Taiwan

Tajikistan

Tanzania

Thailand

Timor-Leste

Togo

Tokelau

Tonga

Trinidad and Tobago

Tromelin Island

Tunisia

Turkey

Turkmenistan

Turks and Caicos Islands

Tuvalu

Uganda

Ukraine

United Arab Emirates

United Kingdom

United States

Uruguay

Uzbekistan

Vanuatu

Venezuela

Vietnam

Virgin Islands

Wake Island

Wallis and Futuna

West Bank

Western Sahara

Yemen

Zambia

Zimbabwe

5. Country of study *

Afghanistan

Akrotiri

Albania

Algeria

American Samoa

Andorra

Angola

Anguilla

Antarctica

Antigua and Barbuda

Argentina

Armenia

Aruba

Ashmore and Cartier Islands

Australia

Austria

Azerbaijan

Bahamas, The

Bahrain

Bangladesh

Barbados

Bassas da India

Belarus

Belgium

Belize

Benin

Bermuda

Bhutan

Bolivia

Bosnia and Herzegovina

Botswana

Bouvet Island

Brazil

British Indian Ocean Territory

British Virgin Islands

Brunei

Bulgaria

Burkina Faso

Burma

Burundi

Cambodia

Cameroon

Canada

Cape Verde

Cayman Islands

Central African Republic

Chad

Chile

China

Christmas Island

Clipperton Island

Cocos (Keeling) Islands

Colombia

Comoros

Congo, Democratic Republic of the

Congo, Republic of the

Cook Islands

Coral Sea Islands

Costa Rica

Cote d'Ivoire

Croatia

Cuba

Cyprus

Czech Republic

Denmark

Dhekelia

Djibouti

Dominica

Dominican Republic

Ecuador

Egypt

El Salvador

Equatorial Guinea

Eritrea

Estonia

Ethiopia

Europa Island

Falkland Islands (Islas Malvinas)

Faroe Islands

Fiji

Finland

France

French Guiana

French Polynesia

French Southern and Antarctic Lands

Gabon

Gambia, The

Gaza Strip

Georgia

Germany

Ghana

Gibraltar

Glorioso Islands

Greece

Greenland

Grenada

Guadeloupe

Guam

Guatemala

Guernsey

Guinea

Guinea-Bissau

Guyana

Haiti

Heard Island and McDonald Islands

Holy See (Vatican City)

Honduras

Hong Kong

Hungary

Iceland

India

Indonesia

Iran Iraq

Ireland

Isle of Man

Israel

Italy

Jamaica

Jan Mayen

Japan

Jersey

Jordan

Juan de Nova Island

Kazakhstan

Kenya

Kiribati

Korea, North Korea, South

Kuwait

Kyrgyzstan

Laos

Latvia

Lebanon

Lesotho

Liberia

Libya

Liechtenstein

Lithuania

Luxembourg

Macau

Macedonia

Madagascar

Malawi

Malaysia Maldives

Mali

Malta

Marshall Islands

Martinique

Mauritania

Mauritius

Mayotte

Mexico

Micronesia, Federated States of

Moldova

Monaco

Mongolia

Montenegro

Montserrat

Morocco

Mozambique

Namibia

Nauru

Navassa Island

Nepal

Netherlands

Netherlands Antilles New Caledonia

New Zealand

Nicaragua

Niger

Nigeria

Niue

Norfolk Island

Northern Mariana Islands

Norway

Oman

Pakistan

Palau

Panama

Papua New Guinea

Paracel Islands

Paraguay

Peru

Philippines

Pitcairn Islands

Poland

Portugal

Puerto Rico

Qatar

Reunion

Romania

Russia

Rwanda

Saint Helena

Saint Kitts and Nevis

Saint Lucia

Saint Pierre and Miquelon Saint Vincent and the Grenadines

Samoa

San Marino

Sao Tome and Principe

Saudi Arabia

Senegal

Serbia

Seychelles

Sierra Leone

Singapore

Slovakia

Slovenia

Solomon Islands

Somalia

South Africa

South Georgia and the South Sandwich Islands

Spain

Spratly Islands

Sri Lanka

Sudan

Suriname

Svalbard

Swaziland

Sweden

Switzerland

Syria

Taiwan

Tajikistan

Tanzania

Thailand

Timor-Leste

Togo

Tokelau

Tonga

Trinidad and Tobago

Tromelin Island

Tunisia

Turkey

Turkmenistan

Turks and Caicos Islands

Tuvalu

Uganda

Ukraine

United Arab Emirates

United Kingdom

United States

Uruguay

Uzbekistan

Vanuatu

Venezuela

Vietnam

Virgin Islands

Wake Island

Wallis and Futuna

West Bank

Western Sahara

Yemen

Zambia

Zimbabwe

What do you learn/know about a!i"cial intelligence?

1. Do you have Electronic Patient Records (EPR) at your hospital? *

Yes

No

Unsure

1. To what extent do you agree with the following statements? *

Strongly

disagree

Disagree

Agree

Strongly

agree

I feel con3dent in using EPR

I receive suocient training on the use of

healthcare-related software

I know how to code

1. How well do you understand the concept of a!i"cial intelligence (AI)? *

1 2 3 4 5

Not well

Very well

1. How much AI-related teaching have you had? *

None

Some

Extensive

1. How much teaching have you had about applications of AI in medicine? *

None

Some

Extensive

1. In your opinion, how accurate are the following statements? *

Strongly

disagree

Disagree

Agree

Strongly

agree

Arti3cial intelligence will revolutionise

medicine in general

The introduction of AI in medicine will make

practitioners deskilled

The physician and other non-interventional

doctors (e.g diagnostic radiologists and

pathologists) will be replaced in the near

future

Surgeons and other interventionalists (e.g

interventional gastroenterologists and

cardiologists) will be replaced in the near

future

1. To what extent do you agree with the following statements *

Strongly

disagree

Disagree

Agree

Strongly

agree

I am worried about the impact of AI in

medicine

I am excited about the potential impact of

AI in medicine

AI will improve medicine in general

AI should be part of medical curricula

1. Which "ve of the following areas of medicine do you think could be most a#ected by introduction of AI? (Please read all options before picking and select 5) *

Radiology

Pathology

Microbiology

Virology

Interventional radiology

Neurosurgery

Ophthalmology

Cardiothoracic surgery

Urology

Dermatology

Oncology

Neurology

Gastroenterology

Cardiology

Anaesthesiolgy

Psychiatry

Infectious disease

Emergency medicine

Electronic patient records (EPR)

Health systems management (including hospital organisation and scheduling)

Adminstration (including note-taking and secretaries) Public health

1. How likely do you think it is that you will be using AI in your work when *

Very unlikely

Unlikely

Likely

Very likely

You graduate

In ten years time

In 20 years time

1. Do you think AI has a place in healthcare in low and middle-income countries? *

De3nitely not

Maybe

De3nitely

1. How will AI be applied to medicine in the future? *

What do you wish to learn about AI?

1. Would you be interested in learning more about AI in your curriculum? *

Yes

No

Maybe

1. Are you interested in learning more about the applications of AI in medicine? *

Yes

No

Maybe

1. To what extent do you agree with the following statements regarding the involvement of doctors in developing AI in medicine? (Don’t answer if you answered “no” to the above question)

Strongly

disagree

Disagree

Agree

Strongly

agree

AI will revolutionise medicine and doctors

should understand this change

If AI does revolutionise medicine, doctors

should be the ones driving this change

If AI does revolutionise medicine, doctors

should at least be involved in making this

change

1. To what extent do you agree with the following statements regarding the nature of AI education? *

Strongly

disagree

Disagree

Agree

Strongly

agree

Medical students should learn about AI at

med school.

Medical Students should be taught how to

use AI in the clinical environment.

Medical Students should be given the

opportunity to learn how to develop AI

algorithms that will revolutionise medicine

Medical students should be exposed to

computer science

1. What would you like to learn about AI that might prepare you for a future in which AI plays an impo!ant pa! in medicine? *

How do we change the medical curriculum to address these learning needs?

1. To what extent do you agree with the following statements regarding the most useful aspects of AI education for medical students? *

Strongly

disagree

Disagree

Agree

Strongly

agree

It is useful for medical students to learn

how to code

It is useful for medical students to learn the

basic principles of AI

It is useful for medical students to learn the

jargon (speci3c language) surrounding AI

It is useful for medical students to meet

computer scientists and collaborate with

them

It is useful for medical students to learn

how to code AI algorithms

It is useful for medical students to start a

technology company (i.e. a tech startup)

1. How can the medical curriculum in your country be changed to address the learning need described in this questionnaire? *

This content is neither created nor endorsed by Google.

# Forms
