# Supplementary figures and images for: Artificial intelligence and medical education: A global mixed-methods study of medical students’ perspectives
Source: Digit Health. 2022 May 2;8:20552076221089099. doi: 10.1177/20552076221089099 (PMC9067043; doi:10.1177/20552076221089099)

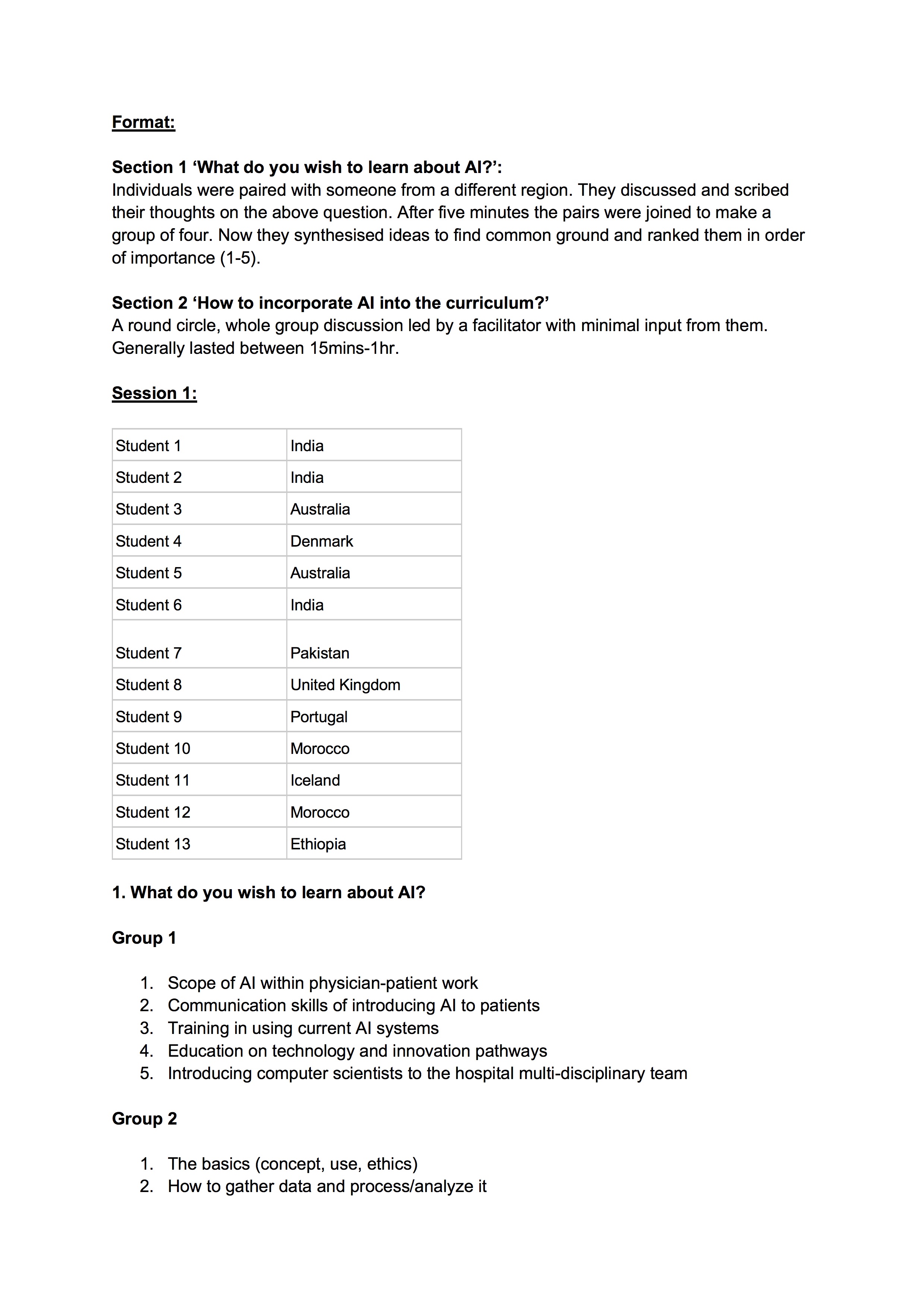

Supplement: sj-jpg-3-dhj-10.1177_20552076221089099 - Supplemental material for Artificial intelligence and medical education: A global mixed-methods study of medical students’ perspectives [file sj-jpg-3-dhj-10.1177_20552076221089099.jpg]
